# Exciton Transfer at Heterointerfaces of MoS_2_ Monolayers and Fluorescent Molecular Aggregates

**DOI:** 10.1002/advs.202201875

**Published:** 2022-06-16

**Authors:** Soyeong Kwon, Dong Yeun Jeong, Chengyun Hong, Saejin Oh, Jungeun Song, Soo Ho Choi, Ki Kang Kim, Seokhyun Yoon, Taeyoung Choi, Ki‐Ju Yee, Ji‐Hee Kim, Youngmin You, Dong‐Wook Kim

**Affiliations:** ^1^ Department of Physics Ewha Womans University Seoul 03760 Korea; ^2^ Division of Chemical Engineering and Materials Science and Graduate Program for System Health Science and Engineering Ewha Womans University Seoul 03760 Korea; ^3^ Center for Integrated Nanostructure Physics (CINAP) Institute for Basic Science (IBS) Suwon 16419 Korea; ^4^ Department of Energy Science Sungkyunkwan University Suwon 16419 Korea; ^5^ Department of Physics Chungnam National University Daejeon 34134 Korea

**Keywords:** contact potential difference, exciton transfer, molecular aggregates, MoS2, photoluminescence

## Abstract

Integration of distinct materials to form heterostructures enables the proposal of new functional devices based on emergent physical phenomena beyond the properties of the constituent materials. The optical responses and electrical transport characteristics of heterostructures depend on the charge and exciton transfer (CT and ET) at the interfaces, determined by the interfacial energy level alignment. In this work, heterostructures consisting of aggregates of fluorescent molecules (DY1) and 2D semiconductor MoS_2_ monolayers are fabricated. Photoluminescence spectra of DY1/MoS_2_ show quenching of the DY1 emission and enhancement of the MoS_2_ emission, indicating a strong electronic interaction between these two materials. Nanoscopic mappings of the light‐induced contact potential difference changes rule out the CT process at the interface. Using femtosecond transient absorption spectroscopy, the rapid interfacial ET process from DY1 aggregates to MoS_2_ and a fourfold extension of the exciton lifetime in MoS_2_ are elucidated. These results suggest that the integration of 2D inorganic semiconductors with fluorescent molecules can provide versatile approaches to engineer the physical characteristics of materials for both fundamental studies and novel optoelectronic device applications.

## Graphical abstract

Integration of blue‐fluorescent organic molecules and atomically thin MoS_2_ monolayers provides energy level alignment appropriate for exciton transfer. Optical characterizations and probe‐based surface charge distribution measurements reveal rapid transfer of photogenerated excitons at the interface. This work highlights a strategy to enable exciton funneling and boosts visible light emission in 2D inorganic semiconductors.

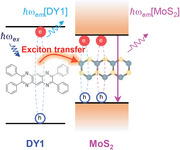

## 1 Introduction

Proper combination of constituent materials is key to tuning the photophysical properties of semiconductor heterostructures, which depend on exciton formation and harvesting at the interfaces.^[^ ^1^, ^2^, ^3^, ^4^, ^5^, ^6^, ^7^ ^]^ Excitons are generated by incident photons in the constituent layers and can be dissociated because of interfacial band offsets. After dissociation, photogenerated electrons and holes in type‐I (II) heterojunctions move in identical (opposite) directions. Thus, spontaneous transfer of photogenerated excitons under light illumination can occur from a high bandgap layer to a low bandgap layer in type‐I heterojunctions.^[^ ^2^, ^3^ ^]^ Such exciton funneling leads to enhanced light emission. Type‐II heterojunctions, in which the transfer of net charge from one layer to the other occurs, are beneficial for the collection of photogenerated carriers in solar cells.^[^ ^4^, ^5^, ^6^ ^]^

Transition metal dichalcogenides (TMDs) are 2D layered inorganic semiconductors that have the form of MX_2_ compounds, in which the transition metal atom, M, is sandwiched between two chalcogen atoms, X. TMDs have attracted growing research attention for optoelectronic device applications since they have sizable bandgap energies (≈1.2–1.8 eV) and optical characteristics tunable by external stimuli, such as strain and electric/magnetic fields.^[^ ^8^, ^9^, ^10^, ^11^, ^12^, ^13^, ^14^, ^15^, ^16^, ^17^, ^18^, ^19^, ^20^ ^]^ TMD‐based heterostructures have been fabricated similar to conventional semiconductors. Energy band structure engineering at heterointerfaces enables control of the electrical transport and recombination of charge carriers in heterostructures.^[^ ^8^, ^9^, ^10^, ^11^, ^12^, ^13^ ^]^ Among numerous TMDs, MoS_2_ has been regarded as one of the strongest candidates for novel optoelectronic device applications.^[^ ^8^, ^9^, ^10^, ^11^, ^12^, ^14^, ^15^, ^16^, ^17^ ^]^

Conjugated organic compounds have emerged as valuable active materials in a variety of devices, such as organic thin film transistors, solar cells, and organic light‐emitting devices (OLEDs).^[^ ^11^, ^12^, ^13^, ^14^, ^15^, ^16^, ^17^, ^18^, ^19^, ^20^, ^21^, ^22^, ^23^, ^24^, ^25^, ^26^, ^27^, ^28^, ^29^, ^30^, ^31^ ^]^ Their utility benefits from the high versatility in the design and synthesis of organic materials. For instance, recent advances have been made in OLEDs by taking advantage of the ability of molecules to accommodate transition metals or to spatially separate frontier orbitals.^[^ ^23^, ^24^, ^25^, ^26^, ^27^, ^28^ ^]^ These molecules are routinely deposited onto semiconductors and metals to form solid‐state devices. Accumulated knowledge indicates that the intrinsic electronic properties of organic compounds are perturbed at interfaces with semiconductors and metals.^[^ ^30^, ^31^ ^]^ Exploitation of the full potential of molecules thus requires an understanding of the electronic processes that occur at the heterojunction between organic aggregates and other materials.

In organic/TMD hybrid systems, the device performance can be improved with the help of synergistic effects of the two materials.^[^ ^1^, ^2^, ^3^, ^4^, ^5^, ^6^, ^7^ ^]^ For example, fabrication of hybrid structures can improve the electrical transport and reliability of OLEDs due to the high carrier mobility and chemical stability of the TMD layers.^[^ ^1^, ^7^ ^]^ Additionally, stacking of organic layers on TMDs is beneficial for improving the light‐matter interaction in TMDs.^[^ ^1^, ^2^, ^3^, ^4^ ^]^ TMDs usually form type‐II band alignment, and charge transfer (CT) rather than exciton transfer (ET) is dominant at the interfaces.^[^ ^9^, ^10^, ^11^, ^12^, ^13^, ^14^, ^15^ ^]^ Insertion of insulating layers between two TMD layers can suppress the interlayer CT process, and hence, the ET process enables enhanced light emission in TMD heterostructures.^[^ ^14^, ^15^ ^]^ Recent works have shown that combinations of suitable materials allow fabrication of both type‐I and type‐II organic/TMD heterojunctions.^[^ ^1^, ^2^, ^3^, ^4^, ^5^, ^6^, ^7^ ^]^ Qiao et al. fabricated heterostructures consisting of MoSe_2_ monolayers (bandgap, *E*_g_: 1.55 eV) and pentacene (*E*_g_: 1.8 eV).^[^ ^2^ ^]^ They reported 3.7‐times‐enhanced photoluminescence (PL) intensity from MoSe_2_ monolayers at the emission wavelength of ≈800 nm. Park et al. fabricated a hybrid structure composed of MoS_2_ monolayers (*E*_g_: 2.11 eV) and perylenetetracarboxylic dianhydride (*E*_g_: 2.55 eV), which exhibited a twofold‐enhanced visible‐light PL yield of MoS_2_ monolayer.^[^ ^3^ ^]^ Since many visible‐light‐fluorescent organic materials are available,^[^ ^22^, ^23^, ^24^, ^25^, ^26^, ^27^, ^28^, ^29^ ^]^ efforts to find materials for type‐I organic/TMD heterojunctions and their careful characterizations should deserve considerable attention for both fundamental science and device applications. Also, systematic investigations to study interfacial excitonic behaviors in such hybrid systems should be established.

We envisioned that understanding the ET and CT processes in heterostructures consisting of fluorescent organic materials and TMDs would provide novel insights into the construction of future devices involving organic/TMD heterostructures. DY1, a blue‐fluorescent molecule recently developed in our group, exhibits excellent photochemical and electrochemical stabilities.^[^ ^29^ ^]^ DY1 is based on a planar pyrazinoquinoxaline core; the planar structure enables the formation of self‐assemblies of DY1. The highly symmetrical molecular structure is anticipated to facilitate the formation of molecular films of DY1 on flat substrates. The propensity for intermolecular packing and lateral layering would provide a valuable opportunity to investigate the behavior of molecular excitons at heterojunctions. In this work, we prepared fluorescent molecular aggregates of DY1 on MoS_2_ monolayer flakes to form hybrid heterostructures. Steady‐state photophysical measurements were carried out to investigate the interfacial excitonic processes at the DY1/MoS_2_ interface. Light‐induced contact potential difference (CPD) changes were studied to examine the photoinduced modifications of the surface charge distribution at the heterointerface. Furthermore, femtosecond transient absorption techniques were employed to study the dynamics of the photogenerated excitons at the heterojunction

## 2 Results and Discussion

**Figure 1:**
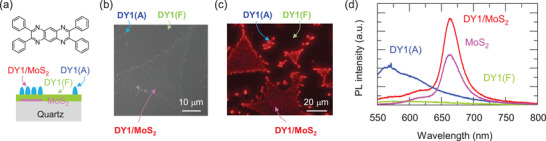
a) Chemical structure of a single DY1 molecule, and cross‐sectional schematic diagram of DY1/MoS_2_/quartz with DY1(A), DY1(F), and DY1/MoS_2_ regions. b) Top‐view SEM image and c) wide‐field PL image of DY1/MoS_2_/quartz. A white LED source with a short‐pass filter (*λ* < 550 nm) and a camera with a long‐pass filter (*λ* > 600 nm) were used for PL imaging. d) Micro‐PL spectra of DY1/MoS_2_/quartz and pristine MoS_2_ flakes on a quartz substrate obtained at an excitation wavelength (*λ*_ex_) of 532 nm.

**Figure** **1**a shows the chemical structure of the DY1 molecule^[^ ^32^ ^]^ and a schematic diagram of a DY1/MoS_2_ heterostructure on a quartz substrate. MoS_2_ monolayer flakes were grown on quartz substrates by chemical vapor deposition (CVD) (Figure S1, Supporting Information),^[^ ^18^ ^]^ and then, DY1 layers were thermally evaporated on top of MoS_2_/quartz.^[^ ^32^ ^]^ A top‐view scanning electron microscopy (SEM) image (Figure 1b) shows that flat films and sparsely spaced aggregates of DY1 are found on the quartz substrate. The thickness of the flat DY1 films was estimated to be 40 nm by a surface profilometer. In sharp contrast, the DY1 molecules preferentially form aggregates on top of the MoS_2_ flakes. The height of the DY1 aggregates, measured by atomic force microscopy (AFM), is as high as several tens of nm (Figure S2, Supporting Information). The density of the DY1 aggregates on the MoS_2_ flakes is much higher than that on the bare quartz surface. The preferential aggregation on the MoS_2_ surface can be attributed to the strong *π*‐*π* interaction among vertically aligned DY1 molecules on the low‐surface‐energy MoS_2_ flake.^[^ ^29^, ^32^ ^]^ The DY1 layers on the quartz substrate with and without aggregates are denoted “DY1(A)” and DY1(F)’. The DY1‐coated MoS_2_ flake is called “DY1/MoS_2_,” which contains high‐density DY1 aggregates.

Figure 1c shows a wide‐field PL image of DY1/MoS_2_/quartz obtained using a bright‐field optical microscope (LV100, Nikon).^[^ ^33^ ^]^ A white LED source (SOLIS‐3C, Thorlabs) with a 550‐nm short‐pass filter was used as an excitation source, and the emission image was taken by a color camera (DS‐Fi3, Nikon) with a 600‐nm long‐pass filter. The PL intensity of DY1(F) is weaker than that of DY1(A) due to the aforementioned difference in thickness. In the case of DY1/MoS_2_, the PL intensity near the MoS_2_ flake edge is much stronger than that in the other regions. As shown in the SEM and AFM images (Figure 1b and Figure S2, Supporting Information), the density of the DY1 aggregates is similar at the edge and in the central region of DY1/MoS_2_. Thus, the PL results indicate that the atomically thin MoS_2_ flakes are capable of quenching the PL of the much thicker DY1 aggregates on them.

Figure 1d shows the micro‐PL spectra of DY1(A), DY1(F), and DY1/MoS_2_ regions in DY1/MoS_2_/quartz and MoS_2_ flakes on quartz substrates obtained with an excitation wavelength (*λ*_ex_) of 532 nm (2.33 eV). The beam diameter of the excitation laser light was ≈1 µm, which was small enough to collect the spectra from selected regions: DY1(A), DY1(F), and DY1/MoS_2_. The PL spectrum of DY1(A) exhibits significantly distinct features compared with that of DY1/MoS_2_. DY1(A) and DY1(F) exhibit very broad emission. The PL intensity of the former is higher than that of the latter due to the aforementioned difference in their thickness. DY1/MoS_2_ and the pristine MoS_2_ flakes show strong emission peaks at a wavelength (*λ*) of 670 nm, which are attributed to the A exciton resonance of the MoS_2_ monolayers (bandgap energy: 1.8 eV).^[^ ^16^, ^17^, ^18^, ^19^ ^]^ The PL intensity of DY1/MoS_2_ at an emission wavelength (*λ*_em_) of 570 nm, where a broad peak appears for DY1(A), is only 20% of that of DY1(A), which reveals that the atomically thin MoS_2_ monolayers can drastically suppress the light emission from the DY1 aggregates. In sharp contrast, the MoS_2_ excitonic resonance peak at *λ*_em_ = 670 nm of DY1/MoS_2_ is 1.7 times larger than that of the pristine MoS_2_ flake, even though the DY1 layer on the MoS_2_ flake reflects and absorbs part of the incident excitation light. The differential reflectance measurements show that the emission spectrum of DY1 overlaps with the absorption spectrum of MoS_2_ (Figure S3, Supporting Information). Therefore, the enhanced emission from MoS_2_ and the suppressed emission from DY1 are likely due to electronic interactions across the DY1/MoS_2_ heterojunction.

**Figure 2:**
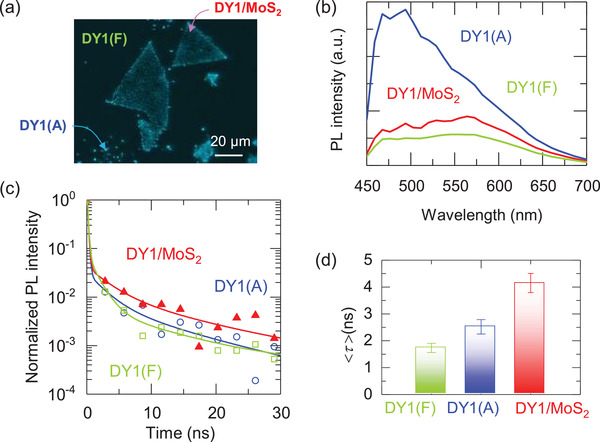
a) Confocal PL scanning image and b) PL spectra of DY1/MoS_2_/quartz at a *λ*_ex_ of 405 nm. The PL image was taken at an emission wavelength (*λ*_em_) of 494 nm. The PL spectra were obtained from the regions in a. c) PL lifetime decay curves obtained from a FLIM image taken at a *λ*_ex_ of 405 nm and a *λ*_em_ of 500 ± 20 nm. Symbols and solid lines correspond to raw data and least‐square fits, respectively. d) Intensity‐weighted average lifetimes, <*τ*>, of DY1(F), DY1(A), and DY1/MoS_2_.

**Figure** **2**a shows the confocal PL scanning image taken at a *λ*_em_ of 494 nm of DY1/MoS_2_/quartz obtained at a *λ*_ex_ of 405 nm (3.06 eV) using a confocal laser scanning microscope (CLSM). The laser beam was focused on the sample surface and the emission spectra at specific pixels in the CLSM image were reconstructed from the PL intensity recorded at each channel of a 32‐channel detector. Typical PL spectra of the distinct sample regions are shown in Figure 2b. As discussed above, the emission from the MoS_2_ monolayers is too weak to be observed at a *λ*_ex_ of 405 nm,^[^ ^16^ ^]^ and hence, the exciton emission from the MoS_2_ monolayer is hardly seen in Figure 2a,b. Substantial PL quenching is found for DY1/MoS_2_ relative to DY1(A). This result is qualitatively consistent with the PL spectra obtained under a *λ*_ex_ of 532 nm (Figure 1d). The PL intensities of the major (*λ*_em_ = 500 nm) and minor (*λ*_em_ = 570 nm) emissions of DY1/MoS_2_ remain at only 70% and 40% of those of DY1(A), respectively. The greater decrease in the major peak can be attributed to the conformeric heterogeneity or the incomplete aggregate formation of DY1 molecules.^[^ ^32^ ^]^ The CSLM image in Figure 2a shows the spatial distribution of the emission intensity from DY1 since the *λ*_em_ of 494 nm is very close to the major emission peak of DY1 (Figure 2b). The emission from DY1 near the edge of the MoS_2_ flake is much stronger than that in the middle of the flake, which clearly reveals the suppression of the light emission from DY1 at the DY1/MoS_2_ interface. The MoS_2_‐induced spectral modification can be clearly shown in the normalized PL spectra (Figure S4, Supporting Information): the PL spectrum of DY1/MoS_2_ near the edge of the flake is similar to that of DY1(A). All these results indicate a notable interaction at the MoS_2_/DY1 interface.

To study the decay characteristics of the DY1 emission, fluorescence lifetime imaging microscopy (FLIM) measurements of DY1/MoS_2_/quartz were performed using scanning confocal microscopy and a picosecond 405‐nm‐wavelength laser, as shown in Figure 2c,d. The PL lifetime was recorded for each pixel in a FLIM image (Figure S5, Supporting Information), and the decay curve was obtained for each region in DY1/MoS_2_/quartz (Figure 2c). The emission intensity of the MoS_2_ monolayer at a *λ*_em_ of ≈500 nm (*λ*_ex_ of 405 nm) is negligibly small, which enables estimation of the intensity‐weighted average lifetime, 〈*τ*〉, ofthe DY1 emission. As shown in Figure 2d, the 〈*τ*〉 of DY1/MoS_2_ (4.2 ns) is longer than those of DY1(A) (2.5 ns) and DY1(F) (1.7 ns) (Table S1, Supporting Information). The extended 〈*τ*〉 of DY1/MoS_2_ is presumably due to the kinetic avoidance of biexcitonic annihilation of DY1 aggregates through the transfer of DY1 excitons to MoS_2_.^[^ ^34^, ^35^ ^]^

**Figure 3:**
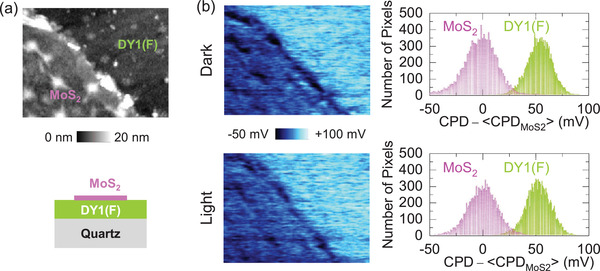
a) AFM topographic image and cross‐sectional schematic diagram of MoS_2_ flakes transferred onto a quartz substrate with evaporated DY1. b) Maps and histograms of [CPD −  〈CPD_MoS2_〉] in the dark and under light illumination (*λ*_ex_ = 405 nm and power density = 8 mW cm^−2^). 〈CPD_MoS2_〉 is the average CPD value measured on the MoS_2_ flake. All the AFM and CPD images have an area of 2 × 1 µm^2^.

For CPD measurements, MoS_2_ monolayers were transferred onto a DY1/quartz sample, as illustrated in the schematic diagram in **Figure** **3**a, and the detailed sample preparation procedures are described in the Experimental Section. Considering the thicknesses of DY1(F) and DY1(A), the distance between the top surface of DY1(A) and the underlying MoS_2_ flake can be as large as 100 nm in DY1/MoS_2_. Even though light‐induced CT occurs at the interface, the resulting CPD change at the DY1/MoS_2_ surface could be too small to be measured due to the electric field screening. An AFM image near the edge of a MoS_2_ flake on a DY1 surface is shown in Figure 3a. The peak‐to‐valley height difference in the AFM image is less than 10 nm, and bumps at the surface are small‐sized aggregates of DY1 molecules. The scanned region in Figure 3a does not have aggregates with a height of several tens of nm, and hence, this region is indicated as DY1(F). The flat surface of DY1(F) enables reliable light‐induced CPD measurements at the MoS_2_ surface without serious concerns about the topographic artifacts. The CPD maps and histograms obtained from the region in Figure 3a in the dark and under 405‐nm light illumination are shown in Figure 3b. The 405‐nm light source was aligned to illuminate the sample area under the Kelvin probe force microscopy (KPFM) tip with an incident angle of ≈60° to avoid blocking of the incident light by the cantilever (Figure S6, Supporting Information). The measured CPD indicates the local electric potential of the sample surface.^[^ ^32^, ^36^ ^]^ Thus, the CPD measurements enable nanoscale mapping of light‐induced changes in the surface potential and charge distributions. The polarity and magnitude of the light‐induced CPD changes can reveal the types and amount of transferred charges.^[^ ^32^, ^36^ ^]^ The results in Figure 3b show that the average CPD at DY1 is larger than that at MoS_2_ in the dark. This relative CPD difference between MoS_2_ and DY1 (50 mV) is almost invariant under light illumination. This suggests that charging by exciton scission does not occur at the DY1/MoS_2_ heterointerface. Consequently, there is no signature of light‐induced CT at the DY1/MoS_2_ interface.

**Figure 4:**
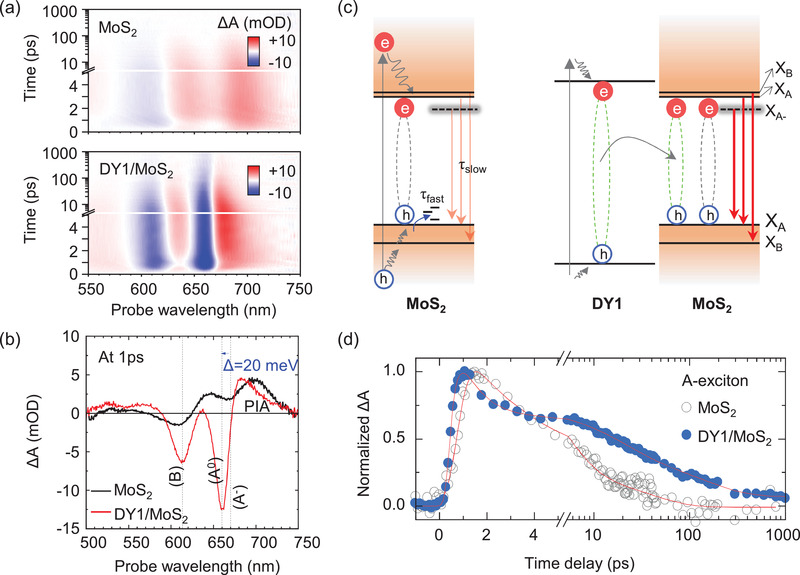
a) Spectrally and temporally resolved TA maps of pristine MoS_2_ (top) and DY1/MoS_2_ (bottom) obtained at a pump wavelength of 450 nm. b) TA spectra of MoS_2_ and DY1/MoS_2_ at a 1 ps time delay. c) Illustration of the photophysical processes in MoS_2_ and DY1/MoS_2_. d) Normalized TA kinetics of MoS_2_ and DY1/MoS_2_ probed at 670 and 660 nm, respectively.

To further clarify the electronic interaction at the DY1/MoS_2_ heterojunction, femtosecond transient absorption (TA) spectroscopy was performed at a pump wavelength of 450 nm (2.76 eV) and an excitation fluence of ≈30 µJ cm^−2^. As written above, DY1/MoS_2_ indicates the DY1‐coated MoS_2_ flake, containing high‐density DY1 aggregates (see Figure 1a,b). The TA signal reflects the density of photoexcited carriers. Spectrally and temporally resolved maps of TA changes (Δ*A*) were obtained from pristine MoS_2_ and DY1/MoS_2_ (**Figure** **4**a). The TA map of DY1/MoS_2_ clearly shows two negative absorptions (photobleaching, PB) peaks of the B exciton at 610 nm and the A exciton at 660 nm. In pristine MoS_2_, the PB peak of the B exciton is observed at 610 nm, but the PB peak of the A exciton is mixed with the broadband of positive absorption (photoinduced absorption). This can be attributed to the excitonic transitions via band filling. The position of each peak in the TA map is similar to that in the PL spectra (Figure 1d). In following discussions, we do not consider ET from the triplet state of DY1 to MoS_2_, since such transfer is impossible due to the spin‐conservation nature.

Figure 4b shows the TA spectra of MoS_2_ and DY1/MoS_2_ at an ≈1 ps time delay. In DY1/MoS_2_, the two exciton peaks are narrower, and their intensity is significantly increased. Moreover, the A exciton peak of DY1/MoS_2_ exhibits a blueshift of 20 meV compared to that of pristine MoS_2_, as marked by the blue arrow in Figure 4b, which indicates an increase in neutral excitons in MoS_2_.^[^ ^37^ ^]^ With 2.76 eV (450 nm) of the excitation photon energy, free carriers can be generated in MoS_2_. The free carriers in MoS_2_ form neutral excitons (A^0^) and trions (A^–^), of which TA signals are located near the energy around 1.9 eV (650 nm) and below, respectively. The TA spectrum of pristine MoS_2_ (Figure 4b) shows that the broader photoinduced absorption (PIA) from A^–^ is dominant compared to the narrower PB signal from A^0^. If CT dominantly occurs at the interface of DY1/MoS_2_, the charged carriers will induce the large A^–^ signal instead of the A^0^ signal. However, a distinct PB signal for A^0^ and a relatively small portion of PIA signal for A^–^ have been observed in the TA spectrum of our DY1/MoS_2_ (Figure 4b). All these results support ET from DY1 to MoS_2_.

In order to further confirm our claim, we studied the power dependence of the TA spectra of pristine MoS_2_. If light‐induced CT occurs at the DY1/MoS_2_ interface, the illumination should increase the number of free carriers in MoS_2_. In the case of pristine MoS_2_, the PIA signal corresponding to the A^–^ contribution will become broader and stronger while increasing the pump power, as expected (Figure S7, Supporting Information). However, the TA spectrum of DY1/MoS_2_ does not exhibit such features (Figure 4b). This is another evidence of the dominant ET process from DY1 to MoS_2_.

In our earlier work, we carried out scanning tunneling spectroscopy studies, of evaporated DY1 molecules.^[^ ^32^ ^]^ Scanning tunneling microscopy characterizations directly visualize the electronic states of molecules with an atomic‐scale spatial resolution. The tunneling conductance spectra suggested that the lowest unoccupied (highest occupied) molecular orbital level of the evaporated DY1 molecules can be higher (lower) than the conduction (valence) band edge of the MoS_2_ monolayers.^[^ ^32^ ^]^ Thus, a type‐I heterojunction is supposed to be formed at the DY1/MoS_2_ interface, as illustrated in Figure 4c. Under such energy level alignment, both electrons and holes (i.e., charge‐neutral excitons) can transfer from DY1 to MoS_2_ under light illumination, which agrees with the CPD results (Figure 3b). As a result, the radiative recombination in MoS_2_ can be enhanced, as observed in the PL data (Figure 1d). These findings also suggest that the aforementioned band filling occurs through the ET process from DY1 to MoS_2_.

Figure 4d shows a clear distinction in the TA kinetics of pristine MoS_2_ and DY1/MoS_2_ at 670 and 660 nm (the A exciton positions). Immediately after light illumination, the density of A excitons in MoS_2_ exhibits a more rapid increase for DY1/MoS_2_ than for pristine MoS_2_. A excitons are generally formed after the generation of B excitons via hole cooling in monolayer MoS_2_.^[^ ^38^ ^]^ In DY1/MoS_2_, as described above, the energy is directly transferred from DY1 to MoS_2_ in the form of excitons. The transferred excitons instantaneously occupy excitonic states in MoS_2_. Consequently, the rise time of the A exciton peak for DY1/MoS_2_ (≈0.4 ps) is smaller than that for pristine MoS_2_, confirming the excitonic ET process across the interface (**Table** **1**).

**Table 1:** Fitting parameters for kinetic curves of A excitons of pristine MoS_2_ and DY1/MoS_2_ in Figure 4d. *A_i_* and *t_i_* denote the fractional amplitude and decay constant, respectively

	A exciton			
MoS_2_	*τ*_rise_ = 0.9 ps	*τ*_fast_ = 3.9 ps *A*_fast_ = 73%		*τ*_slow_ = 30 ps *A*_slow_ = 27%
DY1/MoS_2_	*τ*_rise_ = 0.4 ps	*τ*_1_ = 0.5 ps *A*_1_ = 48%	*τ*_2_ = 18 ps *A*_2_ = 25%	*τ*_3_ = 152 ps *A*_3_ = 27%

The decay kinetics for pristine MoS_2_ and DY1/MoS_2_ are fitted with double and triple exponential decay functions, respectively (solid red lines in Figure 4d), as listed in Table 1. For pristine MoS_2_, the extracted decay time constants of *τ*_fast_ ≈ 3.9 ps and *τ*_slow_ ≈ 30 ps are in good agreement with the reported data in the literature.^[^ ^39^, ^40^, ^41^ ^]^ Specifically, ≈73% of the excited population decays with the fast component (*τ*_fast_), which originates from charge trapping mostly by hole trap states.^[^ ^42^, ^43^ ^]^ The slow component (*τ*_slow_), contributing 27% of the decay, corresponds to the radiative recombination time of excitons because the position of the steady‐state PL emission peak well matches the position of the A exciton peak in the TA spectrum (Figure 4b).^[^ ^44^, ^45^ ^]^ An approximately fourfold extended lifetime is observed for the A exciton of MoS_2_ in DY1/MoS_2_ compared to that in pristine MoS_2_, which is additional evidence of the ET process. The slower component (*τ*_3_ ≈ 152 ps), which contributes 27% of the decay, can be explained by radiative recombination.^[^ ^40^ ^]^ At elevated densities of the A exciton via ET from DY1 to MoS_2_, a strong interaction between carriers leads to rapid depopulation of the A exciton (*τ*_1_ ≈ 0.5 ps).^[^ ^35^ ^]^ This faster component, contributing 48% of the decay, can be attributed to the fast Auger scattering. Nonradiative relaxation via electron‐phonon and slow Auger processes are consistent with the component of *τ*_2_ ≈ 18 ps (25%).^[^ ^40^, ^46^ ^]^

## 3 Conclusion

This work successfully demonstrates the transfer of photogenerated excitons from fluorescent DY1 molecules to 2D semiconducting MoS_2_ monolayers. The hybrid heterostructures were fabricated by evaporating DY1 aggregates on CVD‐grown MoS_2_ flakes on quartz substrates. The PL intensity from the DY1 aggregates on the MoS_2_ monolayers was only 20% of that from the DY1 aggregates on the quartz substrate under 532‐nm light illumination. Sub‐nm‐thick MoS_2_ flakes drastically suppressed the light emission from the DY1 aggregates with thicknesses of several tens of nm. In contrast, the MoS_2_ flakes with evaporated DY1 exhibited 1.7‐fold enhanced PL emission compared to pristine MoS_2_ flakes. The negligible light‐induced CPD changes at the DY1/MoS_2_ interface ruled out the interlayer CT process and indicated the formation of a type‐I heterojunction. Femtosecond TA investigations enabled a detailed understanding of the kinetics of the photogenerated excitons in the DY1/MoS_2_ heterostructures. The TA spectra showed a rapid increase in the exciton density and a fourfold extension of the exciton lifetime in the MoS_2_ monolayers. All these results suggested transfer of charge‐neutral excitons from the higher bandgap DY1 aggregates to the lower bandgap MoS_2_ flakes. This knowledge will be instrumental in the design of high‐performance organic/inorganic hybrid devices. Specifically, our results suggest that integration of fluorescent organic materials and TMDs will provide a new strategy to explore emergent physical phenomena and propose novel optoelectronic devices.

## 4 Experimental Section

### Sample Fabrication

DY1/MoS_2_/quartz samples were fabricated and used for most of the optical characterizations in this work. The synthesis of DY1 was previously reported.^[^ ^32^ ^]^ MoS_2_ flakes were grown on quartz substrates by the CVD technique. A bubbler containing ammonium sulfide solution was used as the sulfur precursor.^[^ ^18^ ^]^ DY1 fluorescent molecules were deposited on MoS_2_/quartz using a thermal evaporator (base pressure: 10^–8^ mbar) following a prior report.^[^ ^32^ ^]^ For CPD measurements, MoS_2_/DY1/quartz samples were prepared. The CVD‐grown MoS_2_ monolayers on SiO_2_/Si were transferred onto quartz substrates with evaporated DY1 by polymer‐based capillary‐force‐assisted stamp transfer.^[^ ^47^ ^]^ Only water was used to separate the MoS_2_ flakes from the quartz substrates since the organic molecules of DY1 could be dissolved in organic solvents.

### KPFM Measurements

The surface topography and CPD maps were obtained by an AFM system (XE‐100, Park Systems) with a glove box. All the measurements were carried out in a N_2_ atmosphere to avoid artifacts caused by ambient gas adsorption. Pt/Ir‐coated Si cantilevers were used as the tip, and the work function was calibrated against a highly ordered pyrolytic graphite reference sample. The dark states were characterized after storing the sample for more than 3 h in a light‐blocked glove box. A laser diode (wavelength: 405 nm, power density: 8 mW cm^−2^) was used as a light source to measure the illumination‐induced CPD changes.

### PL Measurements

Micro‐PL spectra under 532‐nm‐excitation were measured by a multifunctional optical microscopy system using a 532‐nm laser with a power of 5 µW and an acquisition time of 1 s (NT‐MDT, NTEGRA Spectra PNL). Under 405‐nm‐excitation, confocal PL scanning images were obtained using a multiphoton CLSM (LSM780 NLO, Zeiss) and PL spectra at specific pixels in the image were acquired with a 32‐channel spectral detector. The time‐resolved PL decay time was measured using an inverted‐type scanning confocal microscope (SP8 FALCON, Leica Microsystems) with a 20× objective lens (beam diameter: ≈660 nm), a picosecond 405‐nm laser source, and a hybrid photon detector. FLIM images, consisting of 512 × 512 pixels, were recorded using a galvo‐stage and the time‐correlated single‐photon counting technique. All data manipulations for the obtained fluorescence decays were performed using Leica suite software (LAS X Ver. 3.5.2).

### TA Measurements

For TA spectroscopy, the fundamental light from a 1‐kHz Ti:sapphire amplifier with a 790 nm center wavelength (Legend Elite, Coherent) was divided into two beams. One was sent to an optical parametric amplifier (TOPAS Prime, Light Conversion) to generate a pump beam with a certain wavelength. The pump wavelength for photocarrier excitation was tuned to 450 nm, with an energy per pulse of 0.1 nJ pulse^−1^ and a pulse duration of ≈30 fs. The other beam was focused onto a nonlinear crystal to generate a broadband white light continuum in the visible range as a probe beam, which was used to detect changes in absorption as a function of the time delay between the pump and probe pulses. TA data were acquired using a confocal TA microscope system with a spatial resolution of ≈2 µm (HELIOS, Ultrafast Systems).

## Conflict of Interest

The authors declare no conflict of interest.

## Supporting information

Supporting Information

## Data Availability

The data that support the findings of this study are available from the corresponding author upon reasonable request.

## References

[1] J. Sun , Y. Choi , J. Choi , Y, S. K. , J.‐H. Park , S. Lee , J. H. Cho , Adv. Mater. 2019, 31, 1803831. 10.1002/adma.201803831 30786064

[2] J.‐W. Qiao , M.‐S. Niu , Z.‐C. Wen , X.‐K. Yang , Z.‐H. Chen , Y.‐X. Wang , L. Feng , W. Qin , X.‐T. Hao , 2D Mater. 2021, 8, 025026.

[3] S. Park , N. Mutz , S. A. Kovalenko , T. Schultz , D. Shin , A. Aljarb , L.‐J. Li , V. Tung , P. Amsalem , E. J. W. List‐Kratochivil , J. Stähler , X. Xu , S. Blumstengel , N. Koch , Adv. Sci. 2021, 8, 2100215. 10.1002/advs.202100215 PMC8224443 34194946

[4] S. M. Obaidulla , M. R. Habib , Y. Khan , Y. Kong , T. Liang , M. Xu , Adv. Mater. Interfaces 2020, 7, 1901197.

[5] T. Zhu , L. Yuan , Y. Zhao , M. Zhou , Y. Wan , J. Mei , L. Huang , Sci. Adv. 2018, 4, eaao3104. 29340303 10.1126/sciadv.aao3104 PMC5766329

[6] S. B. Homan , V. K. Sangwan , I. Balla , H. Bergeron , E. A. Weiss , M. C. Hersam , Nano Lett. 2017, 17, 164. 28073273 10.1021/acs.nanolett.6b03704

[7] K. C. Kwon , T. H. Lee , S. Choi , K. S. Choi , S. O. Gim , S.‐R. Bae , J.‐L. Lee , H. W. Jang , S. Y. Kim , Appl. Surf. Sci. 2021, 541, 148529.

[8] G. Wang , A. Chernikov , M. M. Glazov , T. F. Heinz , X. Marie , T. Amand , B. Urbaszek , Rev. Mod. Phys. 2018, 90, 021001.

[9] N. P. Wilson , W. Yao , J. Shan , X. Xu , Nature 2021, 599, 383. 34789905 10.1038/s41586-021-03979-1

[10] Y. Liu , N. O. Weiss , X. Duan , H.‐C. Cheng , Y. Huang , X. Duan , Nat. Rev. Mater. 2016, 1, 16042.

[11] Y. S. Kim , S. Kang , J.‐P. So , J. C. Kim , K. Kim , S. Yang , Y. Jung , Y. Shin , S. Lee , D. Lee , J.‐W. Park , H. Cheong , H. Y. Jeong , H.‐G. Park , G.‐H. Lee , C.‐H. Lee , Sci. Adv. 2021, 7, eabd7921.33771864

[12] F. Withers , O. Del Pozo‐Zamudio , A. Mishchenko , A. P. Rooney , A. Gholinia , K. Watanabe , T. Taniguchi , S. J. Haigh , A. K. Gein , A. I. Tartakovskii , K. S. Novoselov , Nat. Mater. 2015, 14, 301. 25643033 10.1038/nmat4205

[13] J. Shi , Y. Li , Z. Zhang , W. Feng , Q. Wang , S. Ren , J. Zhang , W. Du , X. Sui , Y. Mi , R. Wang , Y. Sun , L. Zhang , X. Qiu , J. Lu , C. Shen , Y. Zhang , Q. Zhang , X. Liu , ACS Photonics 2019, 6, 3082.

[14] D. Kozawa , A. Carvalho , I. Verzhbitskiy , F. Giustiniano , Y. Miyauchi , S. Mouri , A. H. C. Neto , K. Matsuda , G. Eda , Nano Lett. 2016, 16, 4087. 27324060 10.1021/acs.nanolett.6b00801

[15] Z. Hu , P. L. Hernández‐Martínez , X. Liu , M.‐R. Amara , W. Zhao , K. Watanabe , T. Taniguchi , H. V. Demir , Q. Xiong , ACS Nano 2020, 14, 13470. 32966063 10.1021/acsnano.0c05447

[16] A. Steinhoff , J.‐H. Kim , F. Jahnke , M. Rosner , D.‐S. Kim , C. Lee , G. H. Han , M. S. Jeong , T. O. Wehling , C. Gies , Nano Lett. 2015, 15, 6841. 26322814 10.1021/acs.nanolett.5b02719

[17] Y. Li , J. Shi , H. Chen , Y. Mi , W. Du , X. Sui , C. Jiang , W. Liu , H. Xu , X. Liu , Laser Photonics Rev. 2019, 13, 1800270.

[18] S. Boandoh , S. H. Choi , J.‐H. Park , S. Y. Park , S. Bang , M. S. Jeong , J. S. Lee , H. J. Kim , W. Yang , J. Choi , S. M. Kim , K. K. Kim , Small 2017, 13, 1701306. 10.1002/smll.201701306 28834243

[19] T. Wang , Y. Zhang , Y. Liu , J. Li , D. Liu , J. Luo , K. Ge , J. Phys. Chem. C 2018, 122, 18651.

[20] J. Shi , J. Zhu , X. Wu , B. Zheng , J. Chen , X. Sui , S. Zhang , J. Shi , W. Du , Y. Zhou , Q. Zhang , A. Pan , X. Liu , Adv. Opt. Mater. 2020, 8, 2001147.

[21] J. Mei , Y. Diao , A. L. Appleton , L. Fang , Z. Bao , J. Am. Chem. Soc. 2013, 135, 6724. 23557391 10.1021/ja400881n

[22] M. Kaltenbrunner , M. S. White , E. D. Glowacki , T. Sekitani , T. Someya , N. S. Sariciftci , S. Bauer , Nat. Commun. 2012, 3, 770. 22473014 10.1038/ncomms1772 PMC3337988

[23] J. Kalinowski , V. Fattori , M. Cocchi , J. A. G. Williams , Coord. Chem. Rev. 2011, 255, 2401.

[24] K. Shizu , J. Lee , H. Tanaka , H. Nomura , T. Yasuda , H. Kaji , C. Adachi , Pure Appl. Chem. 2015, 87, 627.

[25] X. Wu , M. Zhu , D. W. Bruce , W. Zhu , Y. Wang , J. Mater. Chem. C 2018, 6, 9848.

[26] Y. Liu , C. Li , Z. Ren , S. Yan , M. R. Bryce , Nat. Rev. Mater. 2018, 3, 18020.

[27] Y. Kondo , K. Yoshiura , S. Kitera , H. Nishi , S. Oda , H. Gotoh , Y. Sasada , M. Yanai , T. Hatakeyama , Nat. Photonics 2019, 13, 678.

[28] S. Kim , H. J. Bae , S. Park , W. Kim , J. Kim , J. S. Kim , Y. Jung , S. Sul , S.‐G. Ihn , C. Noh , S. Kim , Y. You , Nat. Commun. 2018, 9, 1211. 29572485 10.1038/s41467-018-03602-4 PMC5865184

[29] S. Kim , Y. You , Adv. Opt. Mater. 2019, 7, 1900201.

[30] A. Franco‐Cañellas , S. Duhm , A. Gerlach , F. Schreiber , Rep. Prog. Phys. 2020, 83, 066501. 32101802 10.1088/1361-6633/ab7a42

[31] P. Li , Z.‐H. Lu , Small Sci. 2021, 1, 2000015. 10.1002/smsc.202000015 PMC11935847 40212417

[32] S. Kwon , D. Y. Jeong , W.‐S. Chae , K. Noh , P. Devi , L. Colazzo , Y. You , T. Choi , D.‐W. Kim , Sci. Rep. 2021, 11, 16978. 34417488 10.1038/s41598-021-96437-x PMC8379188

[33] E. M. Alexeev , A. Catanzaro , O. V. Skrypka , P. K. Nayak , S. Ahn , S. Pak , J. Lee , J. I. Sohn , K. S. Novoselov , H. S. Shin , A. I. Tartakovskii , Nano Lett. 2017, 17, 5342. 28753319 10.1021/acs.nanolett.7b01763

[34] Y. Luo , H. Shan , X. Gao , P. Qu , Y. Li , B. Li , X. Rong , B. Shen , H. Zhang , F. Lin , Z. Tang , Z. Fang , Nanoscale Horiz. 2020, 5, 971. 32313908 10.1039/c9nh00802k

[35] N. Kumar , Q. Gui , F. Ceballos , D. He , Y. Wang , H. Zhao , Phys. Rev. B 2014, 89, 125427.

[36] J. Song , S. Kwon , B. Kim , E. Kim , L. N. S. Murthy , T. Lee , I. Hong , B. H. Lee , S. W. Lee , S. H. Choi , K. K. Kim , C.‐H. Cho , J. W. P. Hsu , D.‐W. Kim , ACS Appl. Mater. Interfaces 2020, 12, 48991. 33048546 10.1021/acsami.0c14563

[37] S. Adhikari , J.‐H. Kim , B. Song , M.‐H. Doan , M. D. Tran , L. Gomez , H. Kim , H. Z. Gul , G. Ghimire , S. J. Yun , T. Gregorkiewicz , Y. H. Lee , Adv. Mater. Interfaces 2020, 7, 2000835.

[38] T. Goswamin , R. Rani , K. S. Hazra , H. N. Ghosh , J. Phys. Chem. Lett. 2019, 10, 3057. 31117684 10.1021/acs.jpclett.9b01022

[39] D. Lagarde , L. Bouet , X. Marie , C. R. Zhu , B. L. Liu , T. Amand , P. H. Tan , B. Lrbaszek , Phys. Rev. Lett. 2014, 112, 047401. 24580489 10.1103/PhysRevLett.112.047401

[40] H. Wang , C. Zhang , F. Rana , Nano Lett. 2015, 15, 339. 25546602 10.1021/nl503636c

[41] D. Sun , Y. Rao , G. A. Reider , G. Chen , Y. You , L. Brézin , A. R. Harutyunyan , T. F. Heinz , Nano Lett. 2014, 14, 5625. 25171389 10.1021/nl5021975

[42] S. H. Aleithan , M. Y. Livshits , S. Khadka , J. J. Rack , M. E. Kordesch , E. Stinaff , Phys. Rev. B 2016, 94, 035445.

[43] M. D. Tran , J.‐H. Kim , H. Kim , M. H. Doan , D. L. Duong , Y. H. Lee , ACS Appl. Mater. Interfaces 2018, 10, 10580. 29504404 10.1021/acsami.8b00541

[44] L. Wang , Z. Wang , H.‐Y. Wang , G. Grinblat , Y.‐L. Huang , D. Wang , X.‐H. Ye , X.‐B. Li , Q. Bao , A. T. Wee , S. A. Maier , Q.‐D. Chen , M.‐L. Zhong , C.‐W. Qiu , H.‐B. Sun , Nat. Commun. 2017, 8, 13906. 28054546 10.1038/ncomms13906 PMC5227064

[45] H. Wang , C. Zhang , F. Rana , Nano Lett. 2015, 15, 8204. 26535607 10.1021/acs.nanolett.5b03708

[46] H. Shi , R. Yan , S. Bertolazzi , J. Brivio , B. Gao , A. Kis , D. Jena , H. G. Xing , L. Huang , ACS Nano 2013, 7, 1072. 23273148 10.1021/nn303973r

[47] X. Ma , Q. Liu , D. Xu , Y. Zhu , S. Kim , Y. Cui , L. Zhong , M. Liu , Nano Lett. 2017, 17, 6961. 29058919 10.1021/acs.nanolett.7b03449

