# Supplementary material for: Exciton Transfer at Heterointerfaces of MoS2 Monolayers and Fluorescent Molecular Aggregates
Source: Adv Sci (Weinh). 2022 Jun 16;9(23):2201875. doi: 10.1002/advs.202201875 (PMC9376849; doi:10.1002/advs.202201875)
Supplement: Supplementary file 1 — Supporting Information [file ADVS-9-2201875-s001.pdf]

Supporting Information

Exciton Transfer at Heterointerfaces of MoS_2 Monolayers and Fluorescent Molecular Aggregates

Soyeong Kwon, Dong Yeun Jeong, Chengyun Hong, Saejin Oh, Jungeun Song, Soo Ho Choi, Ki Kang Kim, Seokhyun Yoon, Taeyoung Choi, Ki-Ju Yee, Ji-Hee Kim, Youngmin You*, and Dong-Wook Kim**

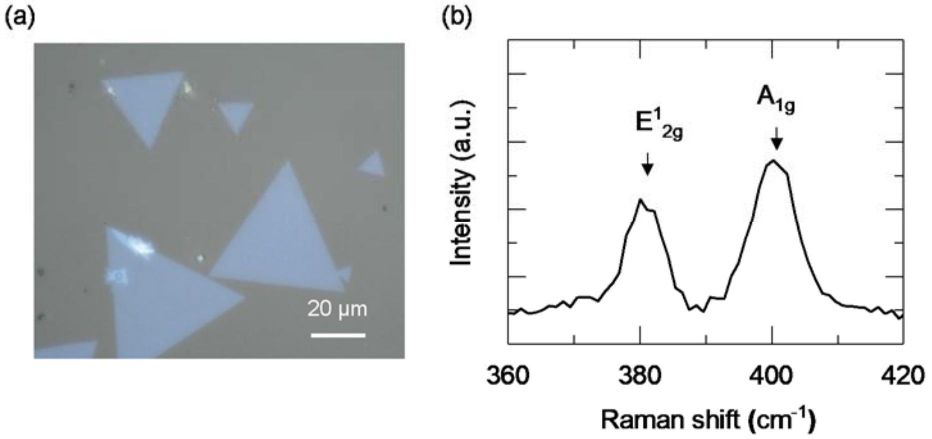

Figure S1. (a) Optical microscopic image and (b) Raman spectrum of a CVD-grown MoS_2 flakes on a quartz substrate. The flakes have triangular shapes with 10-50 μm sizes. The spacing between the two Raman peaks is 19 cm^{-1}, indicating the growth of MoS_2 monolayer flakes.[1]

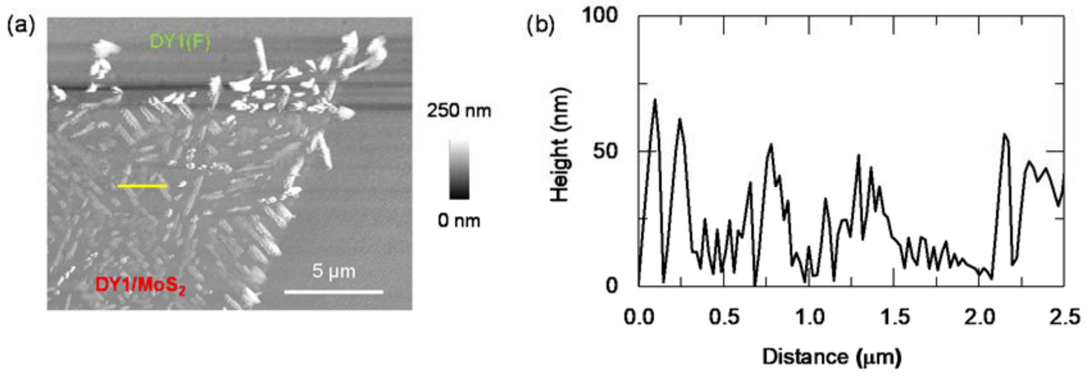

Figure S2. (a) AFM topogrpahic image of the DY1/MoS2/quartz and (b) the height profile of the region indicated by the yellow line in (a).

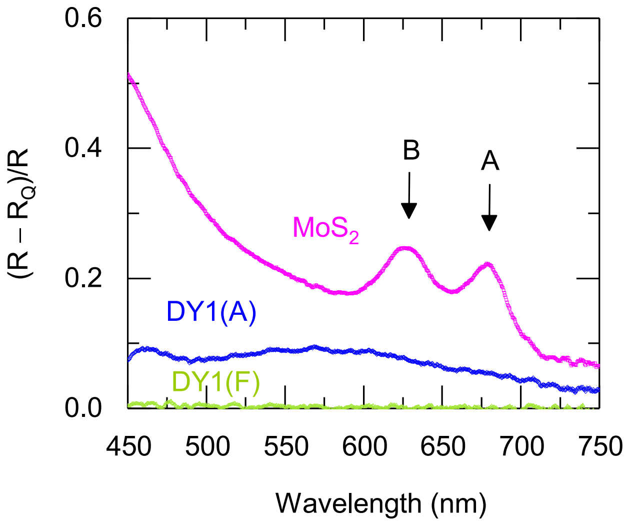

Figure S3. Differential reflectance spectra of DY1(A), DY1(F), and pristine MoS_2, calculated as $(R - R_Q)/R$, where R and R_Q indicate the reflectance of the sample and the quartz substrate, respectively. These spectra were obtained using a home-built micro-reflectance setup equipped with a white LED light source (SOLIS-3C, Thorlabs) and a spectrometer (Maya 2000 Pro, Ocean Optics).[2] The spectrum of MoS_2 exhibits the clear features of the A and B exciton resonances, as indicated in the spectrum. The differential reflectance is proportional to the absorption coefficient and the thickness of the sample.[2] Thus, the spectrum of DY1(A) clearly indicates broad-band absorption in the visible wavelength range.

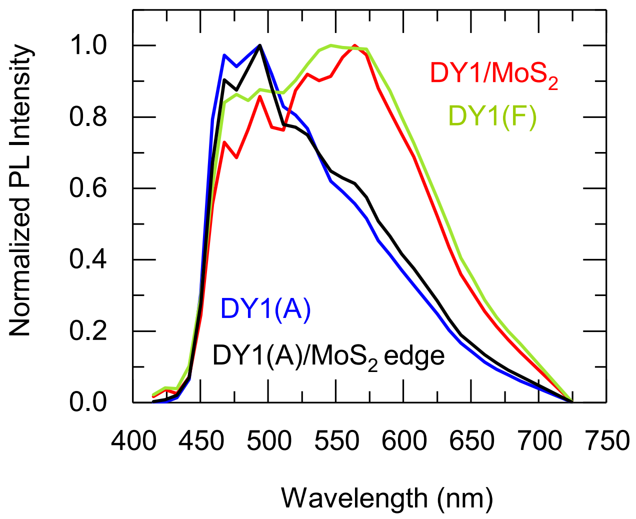

Figure S4. Normalized micro-PL spectra at λ_{ex} = 405 nm in DY1(A), DY1(F), the middle of the DY1-evaporated MoS_2 flake (DY1/MoS_2), and near the edge of the DY1-evaporated MoS_2 flake (DY1/MoS_2 edge). The spectra at DY1/MoS_2 edge and DY1(A) are very similar, whereas they are quite different from that at DY1/MoS_2. This incidates strong interaction at the MoS_2/DY1 interface.

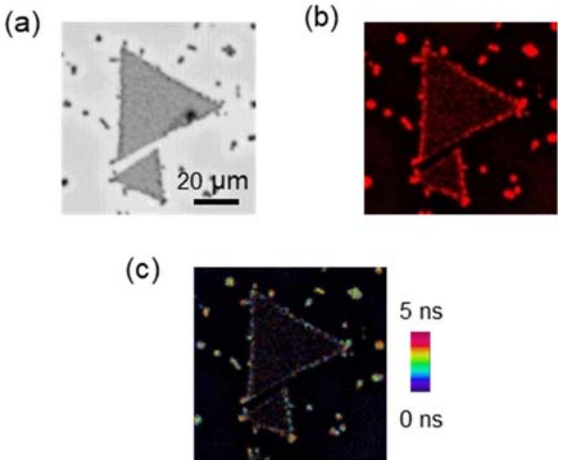

Figure S5. (a) Transmission-mode bright-field optical microscope image, (b) micro-PL image, and (c) FLIM image of DY1/MoS_2/quartz at λ_{ex} = 405 nm and λ_{em} = 500 nm. The color and brightness of the FLIM image represent the lifetime and photon count, respectively.

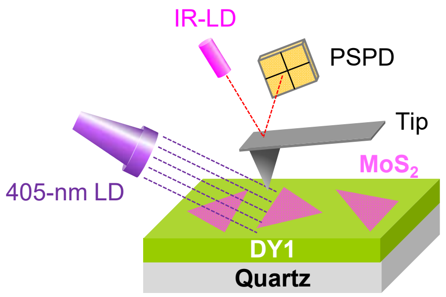

Figure S6. Schematic illustration of our KPFM system. The deflection of the cantilever was detected using an infrared laser diode (IR-LD) and a position-sensitive photodetector (PSPD). An additional laser diode (LD) with the wavelength of 405 nm was used as a light source to measure the light-induced CPD change. The 405-nm LD was aligned to illuminate the sample area under the KPFM tip with an incident angle of ~60°. At smaller angles, part of the incident light was blocked by the KPFM head.

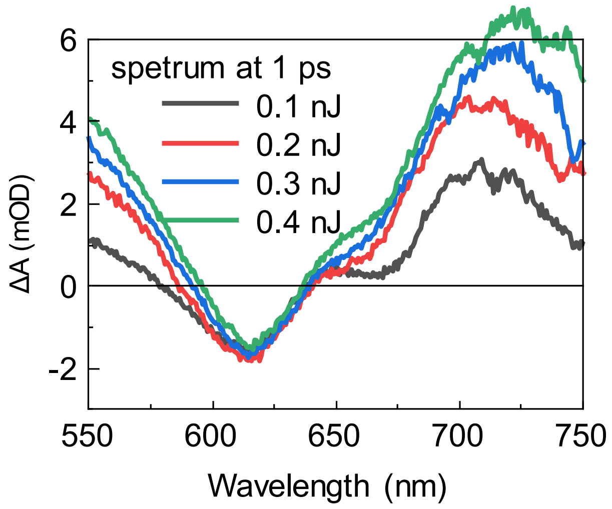

Figure S7. Power dependent TA spectra of pristine MoS_2 probed at 1 ps of time delay (pump power from 0.1 nJ to 0.4 nJ with a step size of 0.1 nJ).

Region				$<\tau>$ (ns)
DY1(F)	τ_1 = 0.17 ns<br>A_1 = 95.50%	τ_2 = 1.43 ns<br>A_2 = 4.19%	τ_3 = 8.34 ns<br>A_2 = 0.02%	1.7
DY1(A)	τ_1 = 0.15 ns<br>A_1 = 98.29 %	τ_2 = 2.45 ns<br>A_2 = 1.31%	τ_3 = 10.85 ns<br>A_2 = 0.40%	2.5
DY1/Mo<br>S_2	τ_1 = 0.16 ns<br>A_1 = 97.38 %	τ_2 = 3.07 ns<br>A_2 = 1.84%	τ_3 = 12.3 ns<br>A_2 = 0.79%	4.2

Table S1. The emission kinetics of DY1 at three regions – DY1(F), DY1(A), and DY1/MoS_2 – were studied. The normalized PL intensity, $I(t)$, was fitted by a triple exponential decay function: $I(t) = \sum_{i=1-3} A_i e^{-t/\tau_i}$, where A_i and τ_i indicate the fractional amplitude and decay constant, respectively. The intensity-weighted average lifetime, $\langle\tau\rangle$, was calculated by $\langle\tau\rangle = \sum \frac{A_i \times (\tau_i)^2}{A_i \times \tau_i}$.

[1] M. Placidi, M. Dimitrievska, V. Izquierdo-Roca, X. Fontané, A. Castellanos-Gomez, A. Pérez-Tomas, N. Mesters, M. Espindola-Rodriguez, S. Lopez-Marino, M. Neuschitzer, V. Bermudez, A. Yaremko, A Pérez-Rodriguez, *2D Mater*. **2015**, *2*, 035006.

[2] R. Frisenda, Y. Niu, P. Gant, A. J. Molina-Mendoza, R. Schmidt, R. Bratschitsch, J. Liu, L. Fu, D. Dumcenco, A. Kis, D. P. D. Lara, A. Castellanos-Gomez, *J. Phys. D: Appl. Phys*. **2017**, *50*, 074002.
